# Current status of pesticide effects on environment, human health and it’s eco-friendly management as bioremediation: A comprehensive review

**DOI:** 10.3389/fmicb.2022.962619

**Published:** 2022-08-17

**Authors:** Vinay Mohan Pathak, Vijay K. Verma, Balwant Singh Rawat, Baljinder Kaur, Neelesh Babu, Akansha Sharma, Seeta Dewali, Monika Yadav, Reshma Kumari, Sevaram Singh, Asutosh Mohapatra, Varsha Pandey, Nitika Rana, Jose Maria Cunill

**Affiliations:** ^1^Department of Microbiology, University of Delhi, New Delhi, India; ^2^Department of Pharmaceutical Sciences, Gurukul Kangri Deemed to be University, Haridwar, India; ^3^Indian Institute of Technology Bombay, Mumbai, Maharashtra, India; ^4^Department of Microbiology, Baba Farid Institute of Technology, Sudhowala, India; ^5^Allergy and Immunology Section, CSIR-IGIB, New Delhi, India; ^6^Laboratory of Alternative Protocols in Zoology and Biotechnology Research Laboratory, Department of Zoology, Kumaun University, Nainital, India; ^7^Cancer Biology Laboratory, School of Life Sciences, Jawaharlal Nehru University, New Delhi, India; ^8^Department of Botany & Microbiology, Gurukul Kangri Deemed to be University, Haridwar, India; ^9^Multidisciplinary Clinical Translational Research, Translational Health Science and Technology Institute, NCR Biotech Science Cluster, Faridabad, India; ^10^Jawaharlal Nehru University, New Delhi, India; ^11^Food Process Engineering, National Institute of Food Technology, Entrepreneurship and Management, Thanjavur, India; ^12^Department of Bioscience and Biotechnology, Banasthali Vidyapith, Newai Tonk, India; ^13^Department of Environmental Science, Dr. Yashwant Singh Parmar University of Horticulture and Forestry, Solan, India; ^14^Biotechnology Engineering, Universidad Politécnica Metropolitana de Puebla, Mexico, Mexico

**Keywords:** pesticides, water, plants, DNA damage, cancer, allergy, biodegradation

## Abstract

Pesticides are either natural or chemically synthesized compounds that are used to control a variety of pests. These chemical compounds are used in a variety of sectors like food, forestry, agriculture and aquaculture. Pesticides shows their toxicity into the living systems. The World Health Organization (WHO) categorizes them based on their detrimental effects, emphasizing the relevance of public health. The usage can be minimized to a least level by using them sparingly with a complete grasp of their categorization, which is beneficial to both human health and the environment. In this review, we have discussed pesticides with respect to their global scenarios, such as worldwide distribution and environmental impacts. Major literature focused on potential uses of pesticides, classification according to their properties and toxicity and their adverse effect on natural system (soil and aquatic), water, plants (growth, metabolism, genotypic and phenotypic changes and impact on plants defense system), human health (genetic alteration, cancer, allergies, and asthma), and preserve food products. We have also described eco-friendly management strategies for pesticides as a green solution, including bacterial degradation, myco-remediation, phytoremediation, and microalgae-based bioremediation. The microbes, using catabolic enzymes for degradation of pesticides and clean-up from the environment. This review shows the importance of finding potent microbes, novel genes, and biotechnological applications for pesticide waste management to create a sustainable environment.

## Introduction

Pesticides are chemical compounds that are used to eliminate insects, rodents, fungi, and weeds. They include insecticides, herbicides, nematicides, fungicides, molluscicides, rodenticides, plant growth regulators, and other compounds (Zhan et al., 2020; Bhatt et al., 2021a; Zhang et al., 2021). It is generally used to prevent illnesses spread by vectors, including crop protection, food preservation, and significant roles in commercial as well as food based industrial practices, i.e., aquaculture, agriculture, food processing, and storage (Mieldazys et al., 2015; Sharma et al., 2019). Any living bodies, either animals or plants, which are harmful for human or animals are known as pests. Pesticides are substances that are used to either kill or prevent the growth of pests.

According to the United States Code of Federal Regulations (CFR), a pesticide is any component or mixture of compounds intended for use as a plant regulator, defoliant, or desiccant (United States Environmental Protection Agency, 2004). Pesticides are defined by the Food and Agriculture Organization (FAO) of the United Nations as substance or mixture of substances attended for controlling, preventing, destroying any pest, animal, or human disease causing vectors, undesirable plants, or animal species affecting food production, managing, selling, storage, and transportation (World Health Organization, 2015). Since ancient times, a variety of chemical compounds have been used to control pests. Sulfur compounds are well known example of such insect and mite control pesticides (Gyawali, 2018). Pyrethrum, a plant (*Chrysanthemum cinerariaefolium*) derived pesticide, has been used for over 2000 years (Unsworth, 2010). Salty water and chemical compounds (organics as well as inorganic) were widely used to control pests’ populations until the introduction of dichloro diphenyl trichloroethane (DDT) by Paul Herman Muller in 1939 as a potent pesticide (Abubakar et al., 2020). However, use of DDT is helpful to increasing the food productivity and shelf-life of food products. Thus, the global demand for DDT increased day by day, which opened the door to synthesizing new chemical substances that act as pesticides. DDT was replaced by organophosphates (OPs) and carbamates (CMs) in the United States in 1975 (Barnhoorn et al., 2009).

The global pesticide consumption in 2019 was approximately 4.19 million metric tons, where China was by far the largest pesticide-consuming country (1.76 million metric tons), followed by the United States (408 thousand tons), Brazil (377 thousand tons), and Argentina (204 thousand tons) (Fernández, 2021). In southeast Asia, WHO reported an annual increase in pesticide usage with 20% of developing countries as pesticide-consumers, including Cambodia, Laos, and Vietnam (Schreinemachers and Tipraqsa, 2012; Schreinemachers et al., 2015). India belongs to one of the major pesticide producing countries in Asia, having 90 thousand tons annual production of organochlorine pesticides including benzene hexachloride and DDT (Khan et al., 2010; Pozo et al., 2011). Between 2010 and 2014, the average cost/benefit ratio was 0.645 g of total pesticides per kilogram of crop yield, with an average yearly consumption of 2.784 kg ha^–1^. Japan (18.94 kg ha^–1^) had the greatest average pesticide usage from 2010 to 2014, followed by China (10.45 kg ha^–1^), Mexico (7.87 kg ha^–1^), Brazil (6.16 kg ha^–1^), Germany (5.12 kg ha^–1^), France (4.85 kg ha^–1^), United Kingdom (4.03 kg ha^–1^), United Status (3.88 kg ha^–1^), and India (0.26 kg ha^–1^) (Zhang, 2018).

**FIGURE 1:**
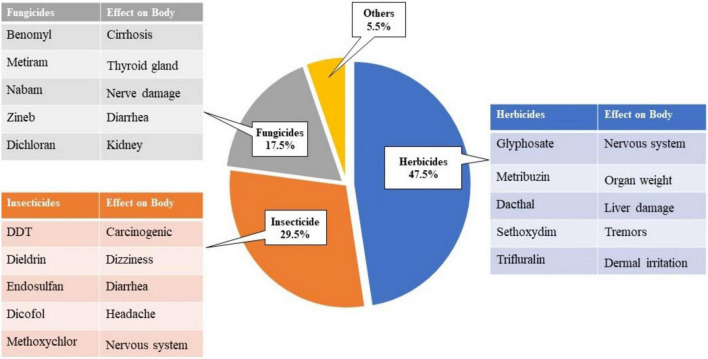
Percentage distribution of pesticides (Nicolopoulou-Stamati et al., 2016; Alengebawy et al., 2021).

Herbicides account for 47.5% of pesticide contributions, followed by insecticides 29.5%, fungicides 17.5%, and other types of insecticides 5.5%, as shown in Figure 1 (Gill and Garg, 2014; Zhang, 2018; Sharma et al., 2019). Pesticides are classified based on a variety of variables. The most often used criteria for pesticide classification are the mode of entry, chemical makeup, and the target it kills. On the other hand, the WHO and Globally Harmonized System (GHS) classified pesticides based on their toxicity or harmful effects, prioritizing public health.

The main advantages of pesticides are the expected immediate gains after application, e.g., eliminating caterpillars, which has the primary benefit of raising cabbage yields and quality. The three major outcomes result in 26 key advantages, ranging from the preservation of recreational grass to the saving of human lives. Secondary benefits are those that arise as a result of the primary advantages but are less obvious or immediate. They might be subtle, less visible at first glance, or long-term in character. As a result, proving cause and effect for secondary benefits is more difficult, although they can still be strong pesticide reasons. Increased productivity of cabbage leads to an increase in economic wealth, which helps to improve children’s health and education systems. Secondary benefits have been identified, including healthier individuals and permanently cultivated land that conserves biodiversity. This accomplishment was aided by the use of high-yield seed types, advanced irrigation technologies, and agricultural herbicides (Bureau, 1993). Similarly, most nations’ productivity and output have increased significantly, such as wheat yields in the United Kingdom and maize yields in the United States. A multitude of factors have been blamed for increased productivity, including better cultivars, machinery use, and fertilizer usage. Pests, insects, diseases, and weeds can substantially reduce the production of harvestable crops; as a result, pesticides have played a crucial role in food production and processing. Warren (1998) also highlighted the huge increase in food production in the United States over the 20th century. Pesticides are used to increase agricultural output and food preservation while ignoring their associated risks. Overuse, exposure, and harmful consequences can all be mitigated by applying it judiciously and utilizing different pesticide categories (World Health Organization, 2009). Many detrimental effects have been seen as a result of widespread pesticide usage, and effective waste management strategies are necessary to address pesticide issues.

**FIGURE 2:**
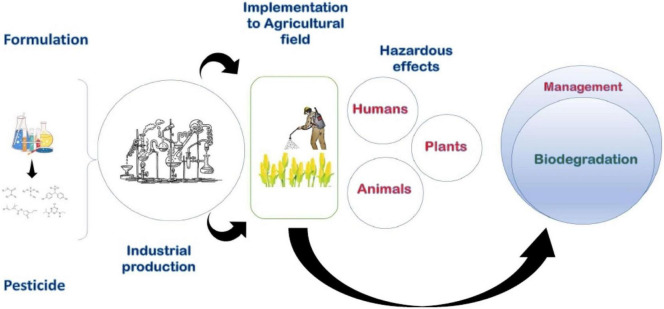
Thematic diagram of the synthesis, production, uses, effects, and eco-friendly management of pesticides.

Pesticide biodegradation is a new way of environmentally acceptable pesticide pollution control for a long-term environmental benefit. Microorganisms play a significant role in the breakdown of pesticides and have been recognized for their influence and many uses in human welfare. Several recent studies have demonstrated the potential of microorganisms, isolated from sewage or soil to degrade pesticides. These microbes include several bacterial and fungal strains, actinomycetes, algae, etc. (Kafilzadeh et al., 2015). The process of pesticide biodegradation, which involves bacteria and enzymes, is described in detail in the biodegradation portion of this review. The entire process of pesticide synthesis or formulation, manufacturing or mass industrial production, detrimental effects on the environment and human health, and biodegradation of pesticides has been shown in Figure 2. To date, there is scant information about the detailed classifications, toxicity, and remediation of pesticides in the environment. Therefore, this review article exploring the new dimensions for removal of pesticides from the environment. This review discusses the impact on living systems, bioremediation approaches, and complete residual removal of pesticides from the environment.

## Classification of pesticides

**FIGURE 3:**
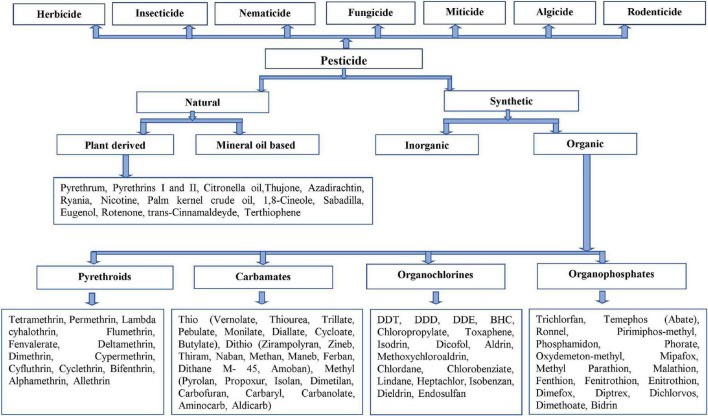
Classification of pesticides (Jayaraj et al., 2016; Hassaan and El Nemr, 2020; Malhotra et al., 2021; Souto et al., 2021; Parra-Arroyo et al., 2022).

The pesticides show the toxicity in the living systems on the basis of their chemical formulations and quantity in an instance. Pesticides are a broad category of products that include antiseptics, disinfectants, anti-bacterial, fungicides, algicides, rodenticides, and herbicides (Garcia et al., 2012). Pesticides are classified into two major categories based on their physical and chemical properties. Pesticide classification by nature of pesticide (synthetic and natural) and acting on pest type is illustrated in Figure 3. Organic chemicals made up the majority of synthetic pesticides, which were grouped into the following four groups: Organophosphates, organochlorines, carbamates, and pyrethroids. Some widely used pesticides and their structures are shown in Table 1. Naturally occurring pesticides, also known as biopesticides, are formed by living creatures such as plants, bacteria, and fungus (Abubakar et al., 2020; Bhatt et al., 2020a, 2021b).

**TABLE 1:** Generally used pesticides and their chemical structures.

Name	Structure	Name	Structure
DDT (Dichlorodiphenyltrichloroethane)		Lindane	
	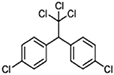		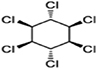
DDD (Dichlorodiphenyldichloroethane)		HCH	
	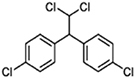		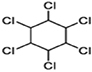
DDE (Dichlorodiphenyldichloroethylene)		Chlordecone	
	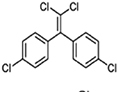		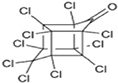
Dieldrin		Toxaphene	
	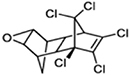		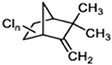
Aldrin		Mirex	
	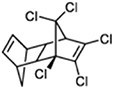		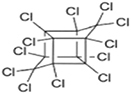
Endrin		Endosulfan	
	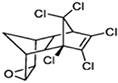		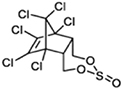
Heptachlor		Chlordane	
	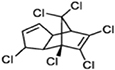		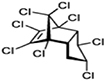

## Classification of pesticides on the basis of toxicity

The amount of pesticides used (dose) and exposure period (time) are the two most important factors for pesticide toxicity that define the acute and chronic toxicity of pesticides. Acute toxicity refers to a pesticide’s toxicity to animals, plants, and humans following a definite short-term exposure of pesticide. A pesticide with a high acute toxicity is fatal, even if only a tiny quantity is absorbed into body. The World Health Organization (WHO) recognizes only acute toxicity for pesticide categorization and based on lethal dosage (LD50) divided into two types, i.e., acute cutaneous (dermal) toxicity (e.g., extremely: less than 50-mg/kg body weight of rat; highly: 50-200-mg/kg body weight of rat; moderately: 200-2,000-mg/kg body weight of rat, etc.) and acute oral toxicity (e.g., extremely: less than 5-mg/kg body weight of rat; highly: 5-50-mg/kg body weight of rat; moderately: 50-2,000-mg/kg body weight of rat, etc.) are shown in Table 2 (World Health Organization, 2009).

**TABLE 2:** Pesticides classification according to WHO guidelines (World Health Organization, 2009).

Class	LD_50_ of rat		Hazardous level
		
	Dermal	Oral	
Ia	Less than 50 mg/kg body weight	Less than 5 mg/kg body weight	Extremely
Ib	50–200 mg/kg body weight	5–50 mg/kg body weight	Highly
II	200–2000 mg/kg body weight	50–2000 mg/kg body weight	Moderately
III	Above 2000	Above 2000	Slightly
U	5000 or above	5000 or above	Unlikely to present acute hazard

The deadly impact of pesticide exposure that persists over time is known as chronic toxicity. Chronic toxicity of pesticides is a worry for the general population and those who work with pesticides directly because of possible exposure to pesticides. Pesticides are now classified into “WHO Hazard classifications” according to the widely used “WHO Recommended Categorization of Pesticides by Hazard.” Following a change in 2009, such a classification was merged with the “Globally Harmonized System (GHS) Acute Toxicity Hazard Category” shown in Table 3 (Mieldazys et al., 2015). Pesticides are also classified based on pest type, mode of action, and disease management strategies as shown in Table 4. Another type of classification is based on its mode of entry, which is divided into the following five categories: (1) Systemic pesticides (absorbed by animals or plants and transferred to other locations, such as in plants, entering into untreated tissues of roots, stems, or leaves *via* multidirectional movement through the vascular system), (2) non-systemic or contact pesticides (they require physical contact with the pest for their action), (3) stomach toxicants (it enters the digestive tract and is absorbed inside the insect’s body; such toxicants are effective for vector control and are used for mosquito or black fly management by malathion application), (4) fumigants (these pesticides are used as poisonous gases or vapor that enter the pest respiratory system *via* spiracles and kill it), and (5) repellents (it is used to keep pests away from treated objects) (Yadav and Devi, 2017).

**TABLE 3:** Pesticides classification according to the Globally Harmonized System GHS (World Health Organization, 2009).

Category	Classification criteria			
	
	LD_50_ of rat dermal	Hazardous description	LD_50_ of rat oral	Hazardous description
1	Less than 50 mg/kg body weight	Lethal if come in skin contact	Less than 5 mg/kg body weight	Lethal if consumed
2	50–200 mg/kg body weight	Lethal if come in skin contact	5–50 mg/kg body weight	Lethal if consumed
3	200–1000 mg/kg body weight	Toxic if come in skin contact	50–300 mg/kg body weight	Toxic if consumed
4	1000–2000 mg/kg body weight	Harmful if come in skin contact	300–2000 mg/kg body weight	Harmful if consumed
5	2000–5000 mg/kg body weight	Possibly harmful if come in skin contact	2000–5000 mg/kg body weight	Possibly harmful if consumed

**TABLE 4:** Pesticides classification according to pest type, functions, and management strategies.

Type of pesticide	Type of pests	Functions	Pests and disease management	References
Aldicarb	Nematicides	Inhibit nematodes (plants parasites)	Damage tissue *via* oxidative stress, and also binds and inhibits acetylcholinesterase (AChE) (controlling acetylcholine neurotransmitter)	Yadav and Devi, 2017; Hassaan and El Nemr, 2020
	Insecticides	Inhibit insects and other arthropods also		
Atrazine	Herbicides	Destroy weeds and other plants, photosystem-II (PSII)–inhibiting	Use to control grasses and broadleaf weeds in sorghum, corn, and sugar cane crops	Gupta and Crissman, 2013; Yadav and Devi, 2017
Avitrol	Avicides	Chemicals that lethal to small seed-eating birds	Used for population management of certain birds (crows, gulls, cowbirds, blackbirds, starlings, grackles, pigeons, sparrows, red-winged blackbirds)	Yadav and Devi, 2017; NIPHM, 2018; Hassaan and El Nemr, 2020
Azoxystrobin	Fungicides	Kill fungi (blights, rusts, molds, and mildews), azoxystrobin act fungal mitochondrion, binds to cytochrome bc_1_ complex and inhibit electron transport thorough oxidative phosphorylation.	Uses to kill Oomycetes, Ascomycetes, Deuteromycetes, BasidiomycetesAnd it controls disease like apple scab rusts, rice blast, powdery and downey mildew.	Yadav and Devi, 2017; Hassaan and El Nemr, 2020
Benzoxazin	Ovicides	Prevention of mites and insects egg growth	In pest managmeent	Yadav and Devi, 2017; Hassaan and El Nemr, 2020
Bifenazate	Acaricides	Control spiders and mites that feed on plants and animals by altering their growth and development. Target site of Bifenazate is mitochondrial, particularly the Q_*o*_ site of that encoded for cytochrome b	Bifenazate uses as an acaricide on strawberry, flowering plants, and nursery ornamentals	Van Nieuwenhuyse et al., 2012; Hassaan and El Nemr, 2020; Authority et al., 2021
Boric acid	Desiccants	Act on plants by drying their tissues	Use to bed bug control	Hassaan and El Nemr, 2020; United States Environmental Protection Agency [US-EPA], 2022
Copper complexes	Bactericides	Prevent bacteria with greater doses, copper works as a broad-spectrum biocide by interfering with nucleic acids, disrupting enzyme active sites, interfering with the energy transport system, cell membranes integrity disrupted	Copper complexes are used to prevent infection of seedlings from plant pathogens by seed treatment	Yadav and Devi, 2017; Hassaan and El Nemr, 2020
Copper sulfate	Algaecides	Control or kill growth of algae	Alter the algal growth and photosynthesis	Lamichhane et al., 2018; Hassaan and El Nemr, 2020
Dichlorobenzene	Moth balls	Inhibit molds and moth larvae and prevent cloths damage	Commonly used to control moths, molds, and mildew	Yadav and Devi, 2017; Eastmond and Balakrishnan, 2010
Fipronil	Termiticides	Fipronil inhibits termites by acting as a GABA antagonist and leads to excessive CNS excitation and causes death	Used in seed coatings and granular soil treatments to control unwanted arthropods in many kinds of food, horticultural, and turf plants	Cannon and Ruha, 2013; Beasley, 2020; Hassaan and El Nemr, 2020
Methiocarb	Repellents	Repel pest vertebrates and invertebrates by its taste or smell	Use as a seedling bird repellant and also effective against frit fly larvae.	Finch et al., 2014; Yadav and Devi, 2017
Methoprene	Larvicides	Prevents larvae growth	Uses as mosquito larvicide, also effective against horn flies, mushroom flies in compost, dipteran pests of livestocks, nuisance flies, highly selectivity for insects and no acute toxicity is expected in humans	Ramaseshadri et al., 2012; Monteiro and Jurado, 2014; Yadav and Devi, 2017
Metaldehyde	Molluscicides	Prevent mollusk’s (snail’s) usually disturbing growth of plants or crops	Use in vegetables and gardens, to kill slugs, snails, other garden pests	Yadav and Devi, 2017; Hassaan and El Nemr, 2020
Rotenone	Piscicides	Toxic and act on fishes	Uses in fisheries and fish management strategies (where unbalanced population of fish)	Gupta, 2014; Hassaan and El Nemr, 2020
Scytovirin	Virucides	Acts against viruses	Control of viral infections and diseases	Hassaan and El Nemr, 2020
Tebuthiuron	Silvicides	Specific to woody vegetation and act on it	Uses to manage the undesirable plants or unwanted forest species and apply to eliminate trees and brush or “entire forest”	Yadav and Devi, 2017
Trifluromethyl nitrophenol (TFM)	Lampricides	Target larvae of lampreys by uncoupling mitochondrial oxidative phosphorylation and ATP production reduces which ultimately leads to death	TFM used to control invasive sea lamprey (*Petromyzon marinus*)	Birceanu and Wilkie, 2018; Hassaan and El Nemr, 2020

## Migration and behavior of pesticides in the ecosystem

When pesticides are administered to a specific area or plant by a farmer, they have the potential to migrate and degrade into the environment and using indigenous microbial strains and physicochemical factors. They show a variety of effects on non-targeted plants as well as kingdom animalia after entering into the ecosystem (Tudi et al., 2021). Pesticides are degraded in our ecosystem by a variety of physical and microbiological processes, including light, temperature, moisture, oxygen, and microorganisms. Pesticides degrade into new chemical entities called metabolites, which can be hazardous or non-toxic depending on their chemical composition (Liu et al., 2015; Marie et al., 2017). Pesticides and their metabolites are transported from a targeted to a non-targeted area *via* adsorption, leaching, volatilization, or surface runoff (Tudi et al., 2021). Because there is an attraction between soil particles and pesticides in sorption systems (attraction influenced by soil organic matter and soil texture), pesticides linger in the soil for a long period of time and have a harmful effect on the soil and ecosystem (Qin et al., 2014).

## Impact of physical and chemical factors on the transformation of pesticides in soil and water

Physical and chemical properties such as molecular weight, ionizability, lipophilicity, polarizability, and volatility of pesticides decide their behavior and biological activity in soil (Bailey and White, 1970; Pignatello and Xing, 1995; Gevao et al., 2000; Beulke et al., 2004). In general, pesticide fate in a soil ecosystem depends on the abiotic transformation related to its physicochemical properties and also on biological transformation related to the presence of live organisms (Różański, 1992). The physical properties make them resistant, reducing losses while chemical structures determine the persistence of pesticides in soil or the environment. These physical and chemical properties of chemical compounds are linked to their movement in soil and aquatic systems and robustness under adverse conditions (Pereira et al., 2016).

Some crucial processes, including adsorption, degradation, and movement, control the behavior and fate of pesticides in soil. Depending on how the pesticide moves, these processes are further classified into leaching, transmission, runoff, microbial and plant absorption. Pesticide transformations in the soil system may vary. Adsorption processes are based on physical forces such as van der Waals or chemical nature, such as electronic interactions (Gevao et al., 2000). Degradation of the pesticides leads to formation of free and bound residues with some altered molecular structures, which are difficult to extract (Roberts, 1984; Gevao et al., 2000). Through diffusion and volatilization, pesticides can dissipate into the atmosphere and wind or runoff leading to subsequent contamination of water bodies. The physical and chemical properties of soil and pesticides, along with other environmental conditions, are mainly responsible for their adsorption by target and non-target organisms, a phenomenon known as bioaccumulation. Chemical and physical characteristics have an impact on leaching, and vertical downward shifting from soil systems. Through the leaching process, pesticides can reach up to groundwater level, making water vulnerable to pollution. Leaching of pesticide into the groundwater in sufficient quantities can pose a hazardous risk to animal and human health. The soil with a sandy nature and low organic content acted as an unstable holding system and weakly absorbed or persistent compounds were most likely to leach-out easily. The chemical, physical, and biological factors of soil with pesticides applied for agriculture practices may influence the leaching process (Steffens et al., 2013). The various agriculture practices are responsible for pesticides translocation in soil or water and the period of their persistence in that environment can be short or longer for weeks, months, or even years due to a number of factors, which include climate change, texture of soil, pH, temperature, moisture, and the content of mineral and organic compounds (Bailey and White, 1970; Gevao et al., 2000; Gupta and Gajbhiye, 2002). Additionally, the leaching and seepage of chemical compounds depends on their mobility as well as persistence, which increases the risk of water pollution (Pereira et al., 2016).

## Pesticide impact on the natural system

Pesticides safeguard around a third of all agricultural goods globally, yet their extensive usage has negative consequences for ecosystems (Zhang et al., 2011). Pesticides harm and accumulate in more other places than crops due to poor management/mishandling, or a lack of information (misuse and overuse). Label instructions on how to use and safety recommendations such as donning rubber gloves and protecting eyeglasses from exposure are not effectively followed by users (EPA Common cause of pesticide incidents) (Qu et al., 2019). Pesticides have a wide range of effects on non-targeted creatures, resulting in environmental issues (Rosell et al., 2008). In the case of air pollution by persistence organic pesticide (POP), is caused by ground and spray. Pesticides that are semi-volatile in nature adsorbed on aerosol particles. The half-lives of these particles are few days to more than a month, it depends on gas-phase reactivity. POP (which are present in the air) undergo a transformation from their native form to a highly toxic form *via* oxidation and photochemical reactions. The migration of these pesticides (POP) depends on the low solubility in water, climate-weather, temperature and humidity (Woodrow et al., 2018). Current use pesticides (CUPs) are more biodegradable in nature as well as less toxic and persistent as compared to previously used organochlorine pesticides (Chen et al., 2020).

## Pesticide impact on the soil system

Pesticides are generally used to protect the crop, but there are several ways in which they can also contaminate the soil. Some of the common reasons include inappropriate use, a lack of information on how to use them in terms of amount, a high amount of runoff into water bodies, and pesticides that are adsorbed, desorb, and broken down during their passage through soil, and these phenomena are dependent on pesticide properties such as persistence, bio-accumulation, and toxicity. Because of this process, the soils become secondary sources of the pollutants with respect to air soil exchange (Pokhrel et al., 2018). According to the report, in European countries, the distribution of 76 pesticide residues was evaluated in 317 agricultural top soil samples, either they contained one pesticide or more than one (Silva et al., 2019).

The bioavailability of pesticides in the food web, pesticide uptake, toxic kinetics, dispersion, metabolism, and excretion all have an impact on species. Pesticides are used excessively and arbitrarily on various crop species, causing harm to beneficial biota such as microorganisms, honey bees, predators, birds, plants, and small animals (Alengebawy et al., 2021).

## Pesticide impact on the aquatic system

Persistence organic pesticide and CUP pesticides enter into the water bodies through a variety of mechanisms, including atmospheric precipitation, chemical or pesticide manufacturing industries releasing unprocessed chemical waste into running water sources (rivers) and other water bodies, where these pesticides travel for miles and contaminate aquatic or water bodies, negatively impacting aquatic ecosystems (Socorro et al., 2016). These pesticides accumulate and transmit from lower to higher trophic levels in aquatic systems, affecting aquatic flora and fauna directly, from which these pesticides have an impact on human health through intake or other means (Woodrow et al., 2018). Chen et al. (2020) studied the aquatic system of shanghai, China and reported a high concentration of CUP (napropamide, atrazine, and chlorpyriphos).

## Effect of pesticide on water eco system

Water is one of the essential elements for all forms of life on earth. About 71% of the water is covered by the earth’s surface. Groundwater constitutes about 30% of the world’s freshwater resources (Marsala et al., 2020). Groundwater quality is under threat due to fast population growth, urbanization, industrialization agricultural pesticides, and population stress (Jayaraj et al., 2016; Wagh et al., 2020). Pesticides may get into groundwater as a result of agricultural runoff from the field or even direct application. The presence of pesticides in water sources is a cause for worry. Pesticides are a type of hazardous chemical that poses a health risk to humans. In many places in the world, groundwater is the most significant source of drinking water. Pesticide pollution is generated from poorly managed agricultural operations and contaminates the surface and ground water. It reduces the quality of drinking water available (Khatri and Tyagi, 2015).

Among the pesticides, organochlorine pesticides (OCPs) have been widely used across the world to control agricultural pests and vector borne diseases (malaria and dengue). Organochlorine pesticides are non-volatile compounds. The problem with using them is that they linger for a long time in natural systems. The use of these substances in an indiscriminate manner has the potential to affect the environment, drinking water systems, and human health. The OCPs’ exposure over time can result in cancer, birth deformities, neurological impairment, reproductive problems, and immune system disease (Agbeve et al., 2014; Fosu-Mensah et al., 2016).

The entry of pesticides into both ground and surface water should be protected. Surface runoff and leaching carry pesticides into water bodies. These pesticides are taken up by plants in the soil, reduced into different chemical forms, and then leached into groundwater. High rainfall increases the risk of pesticides contaminating water. Pesticides that enter groundwater impair the quality of the water, making it unsafe for human consumption as well as for flora and animals. Eliminating pesticides from groundwater is a challenging process. Pesticides in drinking water have negative consequences for both individuals and the ecosystems. According to WHO, around 1 million people are poisoned acutely because of pesticide contact (Hassaan and El Nemr, 2020). To improve production, pesticides will always be a part of human existence and the environment. For pest management, an Integrated Pest Management (IPM) method should be used, which is meant to cause the least amount of environmental disruption by pesticides.

## Effects of pesticides on aquatic animals

Pesticide exposure does not just harm target creatures; it also affects a variety of non-target organisms, with fish being the most notable one. Acute exposure to several pesticides resulted in the mortality of fish in certain cases, whereas lower exposure to the same chemicals resulted in deadly alterations. In many species of fish exposed to various pesticides, changes in hematological parameters such as red blood cells, white blood cells, or plasma and serum level modifications lead to histological abnormalities affecting the liver, kidneys, gills, muscles, brain, and gut (Tahir et al., 2021). Furthermore, genotoxicity has been documented in numerous cases caused by several pesticides. Fish are the lowest rung of the aquatic food chain; thus, they mirror the state of water quality and contamination. Submissive phenomena allow them to collect and store compounds such as heavy metals and pesticides, allowing contaminants in their environment to be recognized. Fish ingest a higher amount of pesticide-infected algae, phytoplankton, and other aquatic plants, causing toxic toxins to progressively accumulate in the tissues and organs of the fish. A small number of these compounds can be regulated by metabolism, while the rest bio-accumulate in the organs and organ systems of fish. Different pollutants are absorbed by the fish’s gills, skin, and alimentary canal, which then disseminate into various organs and tissues, altering physiological and natural phenomena (Banaee et al., 2011). Because the gills are completely exposed to water, they are the most polluted organs. Toxicants enter the body through the gills, increasing oxygen demand. As a result, monitoring any hazardous stress in the aquatic environment is an important metric (Panigrahi et al., 2014).

The following components of a global bicycle should be addressed when determining the principal pathways of pesticide exposure to aquatic systems and biota: (1) The water column, which is frequently the first to be exposed to pesticides, (2) Algae, mosses, vascular hydrophytes, leaf litter, and branches are examples of organic substrates, (3) Inorganic substrates ranging from fine silt to coarse sand particles (Murthy et al., 2013). Pesticide levels in interstitial water and sediments are often lower than in the water column, and lithic biotopes are typically less polluted than the standing waters. Pesticides have toxic effects on aquatic creatures, including fish, at sub-lethal and deadly dosages (Khafaga et al., 2020).

## Hematological causes by pesticide in fish

Fish hematological research has grown in importance as a reliable and sensitive index for assessing biological and pathological changes caused by natural or anthropogenic factors such as microbial infection or levels of contamination in aquatic sources. As a result, hematological parameters are regarded as a crucial tool for determining the body’s functioning condition in response to various stresses (Ali and Rani, 2009). Pesticides changed the hematological parameters of fish in a relatively short time (Rezania et al., 2018). As a result, the hematologic index may be used to efficiently monitor the health and reaction of fish and aquatic creatures to various toxicants, displaying the ecological position of the environment and a typical way to determine the contaminant’s sub-lethal effects (Pimpao et al., 2007). According to Rios et al. (2002), the blood parameters of fish were altered by several genetical and environmental factors. Pesticides affect a variety of fish characteristics, with a focus on blood parameters.

## Pesticide-induced behavioral changes in fish

In several fish species, including *Tor putitora* and *Cyprinus carpio*, pesticides can cause schooling behavior, mucus formation *via* skin’s goblet cells (sliminess), motionlessness, transformations in migration activities, tumbling toward base, jumping, non-responsiveness with hyperexcitability, irregular activities, greater opercular rate (respiration increases), and modifications in body color. Furthermore, they have the ability to change and disturb aquatic vertebrate swimming behavior, such as that of fish and amphibians, as well as impair their growth rates (Stehle and Schulz, 2015). Pyrethroid exposure, decreased the function of the dopamine active transporter, resulting in unpredictable behavior (Wang et al., 2020).

## Malformations and reproductive disorders caused by pesticides in fish

Pesticides may cause reproductive issues in brown trout (*Salmo trutta*) and in Atlantic salmon (*Salmo salar*) (Jaensson et al., 2007). In addition, additional studies discovered a range of developmental abnormalities in fish exposed to the herbicide (Dawar et al., 2016). Pyrethroids have been found in various studies to be harmful to fish reproductive and early embryonic stages. Pyrethroids such as bifenthrin and permethrin can cause egg proteins (choriogenin and vitellogenin) to be delayed in juvenile fish (Brander et al., 2012). Deltamethrin [second-generation (type II) pyrethroid neurotoxin insecticide] at concentrations of 20 and 40 g/L was shown to be damaging to the development of the swim bladder in zebrafish embryos reported by Wu et al. (2020).

## Common effects of pesticides on fish

Pesticides have been shown to have effects on the activity of acetylcholinesterase (AChE), causing an impact on the neurological system and triggering numerous neurotoxic effects (neurotoxicity) in fish (Sharbidre et al., 2011). Fish species such as *Rhamdia quelen*, *C. carpio*, *Colisa fasciatus, Oreochromis mossambicus*, and *Labeo rohita* are affected by pesticide exposure and have also shown the alteration in AChE activity (Joseph and Raj, 2011). In addition, cypermethrin (CYP) caused neurotoxicity and apoptosis in the *Catla catla* brain (Jindal and Sharma, 2019). Pesticides also harm fish’s endocrine systems (Brodeur et al., 2013). When used in large numbers, these chemical compounds may induce molecular toxicity in fish such as *Cirrhinus mrigala*, *Carassius auratus* (goldfish), and *L. rohita* (Ullah et al., 2014). According to histopathological examinations, they have a negative effect on the endocrine systems of *Oncorhynchus mykiss* and *L. rohita* (Dey and Saha, 2014). Pesticides also cause oxidative stress in *T. putitora*, *Lepomis macrochirus*, *Hoplias malabaricus*, *Oreochromis niloticus*, *Clarias gariepinus*, and *L. rohita* by affecting antioxidant defense enzyme activities and reducing the lipid peroxidation marker malondialdehyde, glutathione-*S*-transferase, glutathione reductase, and glutathione level (Muthukumaravel et al., 2013).

## Effect of chemical pesticides on plants

Nowadays, chemical pesticides are widely used by farmers on agricultural land to control weeds, insects, bacteria, fungus, mollusks, rodents, etc. To combat their needs, an increasing population is demanding more foods. Pesticides are used for better crop production (Tomer, 2013). The pesticide defends crops in agricultural land and also minimizes the risk of damage during post-harvest storage. It is very effective and successful in controlling a number of diseases in plants as well as humans, such as malaria and typhoid, but on the other hand, it decreases the soil quality of agricultural land, which is the reason that their negative effects are kept in mind. In 1960, most of the technologically advanced countries banned or restricted the use of pesticides. Ideally, a synthetic or chemical pesticide must be toxic or lethal to the targeted or non-target species. Because of extensive use of pesticides, the pests and insects are going to develop resistance to transformed pesticides like DDT and escape from it.

## Effect of pesticides on vegetables and fruits

The use of pesticides provides a protective layer against pod infection by other pod-feeding insect pests, but damaged pods may not yield seeds or be of poor quality and unfit for use (Mugo, 1998). The usage of chitosan at an early developmental stage boosted plant growth and development and produced higher seed output in rice and soybeans (Chibu et al., 2002). Similar work has been done by Boonlertnirun et al. (2005) in rice and Rehim et al. (2009) in maize and bean.

## Pesticides impact on plant growth and metabolism

Although all pesticides are designed to eliminate or prevent certain plant or animal species, it is a great deal to know about the increasing biological as well as physiological effects of these chemicals on their target organisms. Simultaneously, there are many advantages and potential risks to the use of agrochemicals. Chemically treated seeds are often exposed to substantially greater chemical concentrations than the mature plants during cultivation, so these benefits are countered by the danger of phytotoxicity. Herbicides suppress or control plant weeds by a variety of mechanisms with biological processes such as photosynthesis activity, mitosis cell division, function of enzymes, root and leaf development, DNA and protein synthesis, cell membrane destruction, or encouraging uncontrolled growth. The use of pesticides involves a variety of enzymatic and non-enzymatic alterations in biochemical and physiological antioxidants that can have an initial effect on plant growth from germination and ultimately affect the production of plant yield, e.g., vegetables, fruits, and seeds (Choudhury, 2019; Yengkokpam and Mazumder, 2020).

## Effect of pesticides on plant growth and development

Plant (crop) growth and development do not proceed normally and lead to growth due to the life cycle of the crop, which increases seed size, dry matter accumulation, food storage material in leaves, stems, fruits and roots (Jan et al., 2012). Despite the fact that plant development is influenced by a variety of environmental, genetic, exogenous, and endogenous variables, as well as hormonal situations. Plant development, on the other hand, is an essential phase in determining their producing capability. Brecke and Duke (1980) introduced glyphosate to reduce leaf dry matter accumulation in *Phaseolus vulgaris* L. Basantani et al. (2011) observed an overall decrease in germination rate, dry weight, and root length of *Vigna radiata* after treatment with glyphosate (10 mm). Mishra et al. found that spraying high quantities of pesticides (dimethoate) shortens root and shoot length. Due to increasing levels, dimethoate concentrations in the root are higher than in the shoot (Mishra et al., 2008). Murthy et al. (2005) conducted similar research on *Glycin max L.*

## Effect of pesticides on plant physiology

In the field of pesticide studies, the plant growth is hampered by pesticide accumulation in plants and causes a variety of metabolic disorders, such as chlortoluron affected the plant photosynthetic electron transport chain mechanism (Fuerst and Norman, 1991; Sharples et al., 1997), and Barry et al. (1990) was observed that the PS II reaction center was disrupted. During the photosynthetic pathway, uracil-type herbicides prevent the hill reaction and photosystem II. Reduction of total chlorophyll as well as chlorophyll a, b, and carotenoid content is increased with the increasing application of fungicide doses to plant leaves (Tort and Turkyilmaz, 2003). Sharma et al. (2018a) stated that employment of herbicide causes noxious effects on plants like necrosis, stunting, burns, chlorosis and twisting of leaves. However, Donald (2004) has observed in his experiment that excessive application of pesticides can cause a major reduction in structural vegetation of diversity. Most scientists have been recorded that use of pesticides adversely affects the plant growth and development (Sharma et al., 2015, 2016a,b,c; Shahzad et al., 2018).

## Effect of pesticides on plant defense systems

The use of pesticides causes oxidative stress due to the formation of reactive oxygen species (ROS), which can finally lead to growth deficiency and reduced efficiency of photosynthesis in plants. Plants improve the toxicity because of pesticides by increasing the activity of their antioxidative defense system, which includes non-enzymatic antioxidants and antioxidative enzymes (Xia et al., 2009; Sharma et al., 2015, 2017a,b,2018b). Plant proteins, chlorophyll pigments, and photosynthetic efficacy are all reduced by oxidative stress (Xia et al., 2006).

## Effect of pesticides on human health

The human body gets exposure to pesticides either directly or indirectly. By using pesticides on crops, humans come in direct contact with them and they affect the skin, eyes, mouth, and respiratory tract, and cause acute reactions such as headache, irritation, vomiting, sneezing, and rashes on the skin. The severity of these pesticides on humans depends upon exposure time and concentration. Generally, pesticides are released from the body in the form of excretion (urinary, biliary, and secretory gland). The consumption of such vegetables and fruits that are grown in pesticide contaminated soil and water used for long-term, accumulation increase the concentration of toxins inside the body organs and causes chronic diseases such as neurotoxicity, cancer, necrosis, asthma, reproductive disorder, cardiac disease, diabetes, etc. (Kalyabina et al., 2021). The quaternary nitrogen compounds such as paraquat are associated with neurodegenerative diseases like Parkinson’s, but their molecular mechanism are still not well known (Franco et al., 2010). Similarly, pesticide group of carbamates inhibits the acetylcholinesterase (AChE) activity and is used as a biomarker of neurotoxicity (Gupta et al., 2016). The cancer problem is caused by the various pesticides, but breast cancer is the most common in all cancer types and is associated with organophosphorus (malathion and parathion) that affect cellular growth and proliferation (Calaf, 2021). Similarly, autoinhibitory M2 muscarinic receptors on parasympathetic neurons that innervate airway smooth muscle are implicated in the case of asthma by organophosphorus (Calaf, 2021). It also reduces fertility and creates genital tract anomalies in both males and females by affecting the action of endocrine hormones, their release timing, and imitating these hormones. According to several studies, organophosphorus reduces paraoxonase activity and increases the risk of coronary artery disease (Kabir et al., 2015). In several African nations, hunger and undernutrition are the most serious concerns.

## Role of pesticides in genetic damage

The DNA is an important biomolecule present in living organisms that carries hereditary information and controls the biological synthesis of proteins and enzymes. It acts as the key molecular target of drugs and environmental chemicals such as pesticides. Pesticides interact with DNA and cause conformational changes that could induce gene mutations and lead to adverse health consequences such as carcinogenesis. The acute effects of such chemically synthesized compounds on human health are generally tested and reported before the market launch of these pesticides (Van der Plaat et al., 2018). However, the long-term effect of chronic exposure to pesticides has become a major concern in the last decade.

Pesticide exposure is of the following three types. (1) Direct occupational: Farmworkers who mix and spray the pesticides in agriculture fields; (2) Direct non-occupational: Rural-resident people who live near agriculture fields; (3) Indirect exposure: People who stay far from agriculture areas but get exposed to pesticides through agriculture products, the food chain and contaminated water. Occupational exposure is the most dangerous one as it is linked to a broad range of immediate effects or diseases such as lung disease and airway obstruction. A study conducted in the Dutch population reflects a significant association between the airway obstruction in farmworkers and the corresponding genomic methylation of 31 CpGs (Van der Plaat et al., 2018). Alteration in the genomic methylation pattern affects the expression and repression of genes.

Paredes-Céspedes et al. (2019) reported a notable increase of %5mC in the CpG sites of the WRAP53α gene, “antisense” gene of the p53, in mestizo urban fumigation sprayers who generally use organophosphate insecticides and pyrethroids. Such genetic modifications could act as carcinogenic agents. Differentially methylated CpGs have been found to be unique to the active ingredients of marketed pesticides such as mesotrione, dicamba, acetochlor, picloram atrazine, malathion, glyphosate, and metolachlor (Hoang et al., 2021). Occupational and non-occupational pesticide exposure, as well as chronic and high pesticide exposure in human beings, lead to altered genomic methylation. Various pesticides, including DDT, vinclozolin, methoxychlor, chlorpyrifos methyl, and organochlorine, have been reported to increase or decrease the epigenetic methylation pattern in human beings (Mahna et al., 2021).

The possible genetic damage initiated by occupational pesticide exposure is much greater than that caused by smoking and alcohol consumption (Nascimento et al., 2022). This points to the commonly unacceptable fact that pesticide exposure is much more dangerous than quitting smoking. The random effect of DNA damage in the pesticide-exposed group is roughly 4.63 times more than in the control-exposed group, according to a meta-analytical evaluation addressing probable DNA damage arising from pesticide exposure to farmers (Nascimento et al., 2022). A total of 42 studies were included in the study, with a total number of individuals 2,885 and 2,543 in the exposed and control groups, respectively. In contrast to previous studies, this study found that DNA damage induced by pesticides was not affected by the usage of personal protective equipment, pesticide type, or an individual’s age and gender.

Non-farm employees who reside near agricultural grounds are exposed to pesticides through passive exposure and are thus at risk of pesticide-induced genetic destruction. Non-occupational exposure to pesticides generally corresponds to a high blood concentration of pesticides and increased DNA damage. The pesticides, being oxidizing in nature induces DNA damage *via* oxidative stress (Doǧanlar et al., 2018). The literature represents that aged people, females, and children are more vulnerable to non-occupational pesticide exposure. Increased micronuclei (MN) numbers, oxidative damage, and strand breaks in DNA were seen in the peripheral blood lymphocytes of toddlers living in pesticide-sprayed areas (Kapka-Skrzypczak et al., 2019).

Non-occupational exposure to pyrethroids, a key pesticide used in agricultural and commercial locations, occurs primarily *via* residues through contaminated air and diet. The presence of pyrethroids metabolites in the human urine, including CDCCA [*cis*-3-(2,2-dichlorovinyl)-2,2-dimethylcyclopropane carboxylic acid], DBCA (*cis*-2,2-dibromovinyl-2,2-dimethylcyclopropane-carboxylic acid), TDCCA [*trans*-3-(2,2-dichlorovinyl)-2,2-dimethylcyclopropane carboxylic acid], and 3PBA (3-phenoxybenzoic acid) provides an indication of non-occupational pesticide exposure. The studies to estimate the effect of non-occupational pesticide exposure on human sperm are generally conducted on men recruited from infertility clinics with normal sperm concentrations. The presence of pyrethroid metabolites in human urine is linked to sperm DNA damage increasing and the quality of semen reduces (Meeker et al., 2008).

A positive association were examined with the medium DNA fragmentation index (M DFI) percentage and CDCCA 450th as well as the percentile of 3PBA 450th and high DNA fragmentation index (H DFI) (Jurewicz et al., 2015). Non-occupational exposure to pyrethroids also increases the risk of sex chromosome disomy in sperm nuclei. Radwan et al. (2015) reported disomy in sperm chromosome YY (3PBA), XY (3PBA, TDCCA), 18 (3PBA, CDCCA), 21 (3PBA), and total disomy (3PBA). Those with higher levels of TDCCA and CDCCA have a consistent increased risk of XY, YY, XX, and disomy in the total sex chromosome (7–30%). Males with higher levels of 3PBA displayed an increased risk of YY disomy (28%), a decreased rate of XY disomy (16%), a decreased total disomy (7%), and an increased chromosome 18 disomy (Young et al., 2013).

In reality, human beings and animals are exposed to multiple pesticides and herbicides simultaneously, which may act independently or interdependently. The pesticides organophosphates (OP) and pyrethroids (PYR) act in synergism to increase the risk of germ cell abnormalities (Figueroa et al., 2019). Earlier, Salazar-Arredondo et al. (2008) also reported the chromatin as well as DNA damage in human spermatozoa caused by *in vitro* exposure to a mixture of various organophosphorus pesticides including CPO (chlorpyrifos-oxon), CPF (chlorpyrifos), DZO (diazoxon) or DZN (diazinon), and MePO (methyl-paraoxon).

The pesticides cause DNA damage by interacting with the DNA backbone in either of three ways (1) Intercalation, (2) Grove binding, and (3) Methylation. Extensive studies have been reported in the literature that show the type of interaction between DNA and pesticides (Table 5). The genetic damage caused by pesticides is generally studied in animal models such as mice or rats. Dinitroaniline herbicide, pendimethalin (PND), causes significant DNA damage in the liver and kidney cells of treated rats. This damage is shown to disturb the oxidative balance and activate apoptosis genes (Ahmad et al., 2018).

**TABLE 5:** Mode of interaction of various pesticides with DNA.

Pesticide	Pesticide group	Mode of interaction	References
Chloridazon or Pyrazon	Organochlorine herbicide	Intercalation via GC region	Ahmadi et al., 2011
Fenitrothion	Organophosphorus insecticide	Partially intercalation via NO_2_ and the C Form conformation	Ahmadi et al., 2013
Permethrin, deltamethrin	Synthetic pyrethroid insecticides	Groove binding and partial intercalation	Ahmadi and Ghanbari, 2014
Methyl Thiophanate	Fungicide	Non-intercalative groove binding via AT region	Saquib et al., 2010
Propyzamide	Herbicide	Intercalation via AT region	Zhang et al., 2015
Edifenphos	Organophosphate pesticide	Electrostatic binding minor groove binding via AT region	Ahmad and Ahmad, 2018
Tau-fluvalinate, flumethrin	Synthetic pyrethroid pesticide	Hydrogen bonding and Van der Waals forces, minor groove binding via AT region	Tao et al., 2016
Dinitramine	Herbicide	Hydrophobic interactions, major groove binding	Daneshmehr et al., 2016
Resmethrin	Synthetic pyrethroid insecticides	Hydrogen bonds and Van der Waals forces, groove binding via GC region	Tao et al., 2015
Pendimethalin	Herbicide	Intercalation via GC region	Ahmad et al., 2016
Organophosphates	Pesticide	DNA methylation	Paul et al., 2018
Organophosphate, pyrethroids	Fumigation insecticide	DNA methylation	Paredes-Céspedes et al., 2019
Endosulfan	Pesticide	DNA hypomethylation	Mahna et al., 2021
Glyphosate	Pesticide	DNA hypermethylation	Mahna et al., 2021
Diazinon	Pesticide	DNA hypermethylation	Mahna et al., 2021
Fonofos, parathion, terbufos	Pesticide	DNA hypermethylation	Mahna et al., 2021

## Pesticides’ role in cancer

Several epidemiological and molecular research highlighted a close association between persistent pesticides exposure and increased risk of diseases such as neurodegenerative disorders, endocrine disruptors, respiratory complications, reproductive disorders, and birth defects (García et al., 2017; Larsen et al., 2017; Addissie et al., 2020; Bast et al., 2021; Bhadauriya et al., 2021; Witczak et al., 2021; Gea et al., 2022; Iteire et al., 2022). In addition, the carcinogenic, teratogenic, and mutagenic nature of these compounds are also believed to be a contributing source of cancer development in the human population.

**FIGURE 4:**
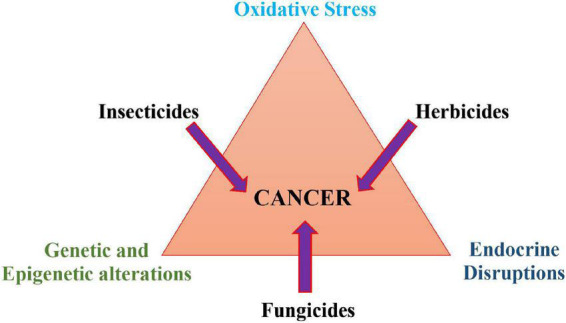
Major molecular mechanisms associated with pesticides-induced carcinogenesis.

It has been observed that a person with a direct exposure to pesticides is highly susceptible to several human malignancies such as cancer including head, neck, breast, thyroid, brain, colorectal, pancreatic, lung, leukemia, prostate, non-Hodgkin lymphoma and ovarian cancer (Obiri et al., 2013; Pardo et al., 2020; Leonel et al., 2021; Lerro et al., 2021). Several pathways have been discovered to date; however, the major molecular mechanism that is likely to cause pesticide-induced carcinogenesis involves oxidative stress, genetic and epigenetic changes, and endocrine disruptions (Figure 4). For instance, excessive production of ROSs as a result of pesticide exposure can disrupt the cellular equilibrium between pro and anti-oxidant molecules and induce oxidative stress to induce macromolecule damage, leading to dysregulation of several fundamental processes and subsequently stimulating cancer initiation, growth, progression, metastasis, and chemotherapeutic resistance (Pardo et al., 2020; Leonel et al., 2021; Lerro et al., 2021).

In a study by Želježić et al. (2018), herbicide terbuthylazine exposure was reported to form reactive terbuthylazine metabolites, which induce DNA cross-links in both *in vitro* and *in vivo* systems. Thakur et al. (2018) reported that oxidative DNA damage induced by two extensively used organophosphate pesticides, monocrotophos and chlorpyrifos, modulate the AP endonuclease 1-dependent base excision repair pathway to promote the proliferation of lung cancer. Similar toxic effects were also observed for widely used insecticide, neonicotinoid (dinotefuran, nitenpyram, and acetamiprid) exposure, which resulted in disturbance of amino acid metabolism, accumulation of lipids, and enhance oxidative stress in ICR mice *via* decreasing glutathione (GSH) level and increasing superoxide dismutase (SOD) level (Yan et al., 2020). Polymorphism in oxidative stress-related genes (catalase, glutathione peroxidase, glutathione-*S*-transferases, manganese superoxide dismutase, and paraoxonase) may not be directly linked to cancer; instead, they make people more vulnerable to pesticide-induced oxidative stress (Kaur et al., 2018; Moradi et al., 2018; Costa et al., 2019; Mbah Ntepe et al., 2020).

Endocrine disruptions are caused by agents/EDCs (endocrine disrupting chemicals) that affect the natural function of the endocrine (hormone) systems of a body by disrupting the synthesis, release, binding, specific activity or abolition of normal hormone, which are responsible for the growth, development, fertility, and homeostasis maintenance of a cell. Pesticides are well known for disrupting endocrine function *via* mimicking or delaying the release of natural hormones, thus, being accountable for decreased fertility, neurological or behavioral dysfunctions, thyroid gland abnormalities, immunosuppression, and carcinogenesis (Kori et al., 2018; Pizzorno, 2018; Requena et al., 2019; Brandt et al., 2020; Montes-Grajales and Olivero-Verbel, 2020). Most of the pesticides work as agonists to activate numerous hormone receptors for instance androgen receptors, estrogen receptors, pregnane X receptors, nuclear hormone receptors, and aryl hydrocarbon receptors (Eve et al., 2020; Lacouture et al., 2022). Low dose of phenolic EDCs upregulated aromatase signaling and thus regulated aromatase-induced 17β-estradiol biosynthesis to support breast cancer cells proliferation (Williams and Darbre, 2019). Furthermore, thiacloprid and imidacloprid exposure stimulates CYP19 promoter activity, which increases estrogen biosynthesis *in vitro* in a similar manner to hormone-dependent breast cancer (Caron-Beaudoin et al., 2018). Recently, antagonistic effects of pesticides have also come into focus. For example, cypermethrin showed an inhibitory effect on the dihydrotestosterone activated interaction of the androgen receptor with its coactivators ARA70 and ARA55 (Zhen et al., 2020). Zhang et al. (2018) discovered a novel mechanism of endocrine disruption, where 16 pesticides showed anti-mineralocorticoid activity, among which 14 interfere with nuclear translocation of the mineralocorticoid receptor to promote hepatocellular carcinoma. Another novel pathway involves fungicides (Prochloraz, vinclozolin, and M2) competing with the androgen receptor, ZIP9, to block pro-apoptotic signaling in prostate cancer cells (Thomas and Dong, 2019). In another study, glyphosate was reported to inhibit aromatase signaling in a non-competitive manner while imidacloprid and thiacloprid inhibited estrogen receptor activity in MELN cells (Zhang C. et al., 2020). Overall, we observed that pesticides can alter the cellular metabolism in multiple ways to induce cancer risk. It was also observed that a person with direct or occupational exposure along with inherent genetic susceptibilities is more prone to disease.

## Pesticide exposure causes allergies and asthma

The salubrious nature of pesticides makes them ideal candidates for modern agriculture techniques and enhanced crop-production. However, extensive usage of pesticides leads to serious health conditions due to their bio-magnification and persistent nature (Sharma et al., 2019). The vapors of pesticides can invade water, soil, air and finally enter the food chain, thereby threatening to human health (Sharma et al., 2017c). It has been found that food contaminated with pesticide residues leads to a higher level of toxicity compared to drinking or inhaling contaminated water or air (Margni et al., 2002). Pesticides can mimic or antagonizes natural hormones, thus disbalancing hormonal homeostasis, reducing immunity, causing cancer and other reproduction-related problems (Yadav et al., 2015).

Studies have reported that acute or chronic exposure to such pesticides leads to airway diseases such as allergic rhinitis or asthma. The population at high-risk of developing health issues due to pesticide exposure includes mainly farm workers, pest control workers, or workers from agricultural industry, and the other environmentally exposed individuals residing near farms or agriculture fields or the individuals exposed to household pesticides (Ernst, 2002; Ndlovu et al., 2011).

More evidence of exposure to pesticides has been reported among farmers and their families along with insecticide producers or applicators across the globe, such as the United States, Canada, France, and Australia, with increased asthmatic conditions (Baldi et al., 2014). Such exposures may lead to decreased FEV 1 (forced expiratory volume in 1 s) of forced breath with exacerbation of asthma and also induction of autonomic function and altered immune response (Osteen and Fernandez-Cornejo, 2013; Henneberger et al., 2014). In relation to the use of domestic pesticides, exposure to insecticides has a particularly important role in the induction and worsening the asthma and asthma-like syndrome (Osteen and Fernandez-Cornejo, 2013). In countries such as the United States, where asthma morbidity is high due to cockroach sensitization, insecticides are used to control exposure, which in turn increases pesticide exposure, and asthma morbidity (Garthwaite et al., 2012).

Another study on farm operators showed a significant association between current asthma and lifetime allergic rhinitis by the use of carbaryl and 2,4-dichlorophenoxyacetic acid. Approximately 40% of 2.1 million farm operators had lifetime allergic rhinitis in 30% farmers and 5.1% has current asthma (Patel et al., 2018). Some synthetic insecticides, such as pyrethroid, used to control mosquitoes are known to cause asthma attacks, while permethrin and Sumithrin are key contributors to headaches, tremors, convulsions, asthmatic attacks, and can be lethal in more serious conditions (EPA et al., 2009; Amaral, 2014). Not much is known about specific pesticides responsible for allergic/asthmatic exposure. Studies from Canada, Spain, India, or South Africa demonstrated that pesticides belonging to class organophosphates and carbamates are particularly involved in causing asthmatic conditions (Hernández, 2015). These studies mainly performed lung function assays such as spirometry, and lung volumes/capacity, but none has involved primary inhalation challenge testing.

## Effect of pesticides on asthma

Pesticide use and asthma incidences were reported in the common people as reported by some of the studies performed in the United States population. The US urban population was found to be chemically intolerant to at least three commonly used chemicals such as paints, pesticides, perfumes, or car exhaust. Subjects reported asthmatic and respiratory symptoms such as shortness of breath with wheezing and chest tightness (Baldwin et al., 1997; Amaral, 2014). A cross-sectional study of US National Health and Nutrition showed an association of residential pesticides with respiratory problems in children, mostly used in the kitchen or dining area (Xu et al., 2012). The incidence of such residential exposures has increased in the United States from 1.1 to 4.4 per million (Amaral, 2014; Hudson et al., 2014). Indoor air pollution, caused by pesticide spraying or the use of over-the-counter insecticides, has exacerbated symptoms such as irritation, lower respiratory pain, wheezing, dyspnea, and dry cough. In a randomized investigation of 25 asthmatic participants exposed to modest amounts of aerosols, asthmatic symptoms worsened when compared to a control group (given water). Asthmatic patients had a more than 15% decrease in FEV1 and severe bronchial responsiveness, with symptoms affecting the chest, nose, and eyes (Salome et al., 2000).

Previously, it has been reported that allergic asthma was relatively more common in children than in adults. The risk of environmental exposure to pesticides was higher for school children, especially those living near farms or rural areas (Matthews, 2005; De Barros Rodrigues et al., 2022). Children with acute symptoms have been reported due to pesticide drift near their schools, or they might be at even higher risk because of accidental contact while playing on agriculture farms with empty containers of contaminating materials (Buralli et al., 2020). In a longitudinal study, children living in agricultural communities had higher amounts of the dialkylphosphate (DAP) metabolite in their urine. The DAP metabolites are general to organophosphorus pesticides and are responsible for the temporal pattern of children’s pesticide exposure upon pesticide spraying in an agricultural region (Koch et al., 2002). Other factors for children’s hospitalization related to pesticide exposure are their increased respiratory rate, comparatively larger surface area of skin, and elevated metabolic rate (Sharma et al., 2019).

A few studies investigated the role of allergic asthma as well as other respiratory symptoms due to pesticide exposure among women. The studies were mainly focused on male workers, associated directly or non-directly with agricultural fields, but it was evident that women are also increasingly affected and at high-risk due to pesticide exposure (Ndlovu et al., 2011). In a study, Hoppin et al. (2008) evaluated pesticide and occupational exposures as risk factors for farm women. Out of 25,000 women with atopic and non-atopic asthma, who grew up on farms and used pesticides, were more likely to develop atopic asthma than the non-users. In an infant’s environmental health birth cohort study of 266 mothers in Costa Rica, by performing a survey, they investigated the outcomes of respiratory and allergic conditions in mothers upon exposure to pesticides and other environmental metabolites. The study found significant association of high asthma score and urinary levels of thiabendazole metabolite in women living near waste burning farms and women living in agriculture farms reported eczema and itch rash (Garry, 2004; Alhanti et al., 2021). Another study linked pesticide exposure to changes in the serum metabolome after eating fruits and vegetables (FVs). The study analyzed 171 women under infertility treatment and showed significant associations of metabolic pathways upon the eating of either high or low-to-moderate pesticide residue FVs. Different biological pathways were associated with the intake of high or low pesticide residues, including metabolism (energy, cellular receptor, enzyme, lipid, and vitamin) and intracellular signaling (Hood et al., 2022). There is a need to perform more such unique studies about associations between environmental and occupational pesticide exposures and respiratory and allergic diseases. Such an insightful study related to dietary intake of pesticides might provide information on potential mechanisms associated with human diseases.

## A link between food allergies and pesticides

Food allergy affects up to 10% of the world population, with more severity in infants as compared to adults. It has been referred to as the “second wave” of the allergy epidemic, following asthma (Loh and Tang, 2018). In parallel, the use of pesticides such as organophosphates has been increased in agriculture and industries. This increased use of organic agents might prolong the allergic manifestations in atopic individuals by potential mechanisms such as epigenetic control of allergen expression, modifying proteins to make them even more allergenic; or increased polyamine production in stressed condition (Falak et al., 2012; Loh and Tang, 2018).

People who are exposed to chemicals either through chlorinated water or come into contact with foods that contain them or breathe polluted air are more likely to develop food allergies. Chemicals like dichlorophenols can alter the microbiota of the human body and in turn influence the body’s immune system to trigger such reactions. In contrary to hygiene hypothesis, dichlorophenols can kill microbes and clear the environment such that young children become prone to developing allergy risks. In an international survey of the United States (NHANES) in the period 2005–2006, 2,200 children aged 6 were checked for dichlorophenol levels in their urine along with allergies to peanuts, eggs, milk, and shrimp. It was found that children with high levels of urine dichlorophenol were 80% more likely to develop allergies (Jerschow et al., 2012).

An ample number of studies have been performed related to pesticide exposure and asthma, but a lot more meticulous studies need to be accomplished. The previous data was generated accordingly self-reported or doctor-diagnosed asthma, which needs to be refurbished with bronchial responsiveness measurements and lung function. To strengthen the data, a detailed molecular and genetic phenotyping must be explored to study the effect of pesticides in different types of asthmatic conditions (Jerschow et al., 2012; Loh and Tang, 2018). Studies on different active and organic ingredients or new formulations along with potent agents might provide important insights, such as between asthma and exposure to pesticides. The recent cohort studies identified certain biomarkers directly linked to pesticide exposure and asthma, thus new biomarkers for the different and generally used pesticides can be considered. More robust measurement of pesticide exposure depending upon the biomarkers should be the focus of the future comprehensive studies. Their metabolic rate, bioactivity, life time, and threshold levels must be recorded to understand the pathophysiology of the underlying asthmatic or atopic conditions. Finally, more longitudinal studies offering a large sample size over a longer period of time can be a big step toward understanding the biological pathways at the gene level that can directly link pesticide exposure to disease development.

## Pesticide effects on preserved food

Pesticides play a global role in the protection, preservation, comfort of food, fiber, and human health (Winteringham, 1971). However, the excessive and uncontrolled use and misuse of pesticides, as well as their long-run transportation and volatility, cause widespread environmental damage or contamination. Moreover, the occurrence of many highly toxic, non-patented, and eco-resistant chemicals creates severe health concerns that causes global impact simultaneously (Ecobichon, 2001). In India, the value addition and processing of ready-to-eat (RTE) or ready-to-serve (RTS) packaged products impact a lot on monitoring the levels of pesticide residues during the final consumption. However, during the processing of raw agricultural commodities (RAC), the levels of pesticides are mostly governed by the concentration level and physico–chemical characteristics of the product to be processed (Muralidhara et al., 2022). Researchers reported that pre- or post-processing steps are capable enough of reducing the load of pesticides in the final product. However, in certain specific cases, processing aids in the accumulation of pesticide residues (e.g., extraction of oil from oil seeds) (Kaushik et al., 2009; Muralidhara et al., 2022). Therefore, a maximum residual limit (MRL) of pesticides needs to be established in the case of food products attaining paramount exposure to pesticides during their pre-harvesting phase (Scholz et al., 2017).

Processing factor (Pf) – During the processing of foods, there is a chance that the whole mass of pesticide residues can be assimilated into processed products. Therefore, the effect of pesticide residues on food products can be expressed by a term “processing factor” and can be calculated as follows.


$$\mathrm{Processing}\,\mathrm{factor}\,\left(\mathrm{Pf}\right)=\frac{\mathrm{Pesticide}\,\mathrm{concentration}\,\mathrm{in}\,\mathrm{raw}\,\mathrm{product}}{\mathrm{Pesticide}\,\mathrm{concentartion}\,\mathrm{in}\,\mathrm{processed}\,\mathrm{product}}$$

The processing factor is an integral tool to generate data for global regulatory authorities monitoring the residual limits and also helps in assessing the risks by estimating the refined dietary exposure of pesticides in a processed food commodity before consumption (OECD, 2008).

## Effect of pesticide residue on processing operations

Processing operations play a significant role in maintaining or lowering the pesticide limit in the final value-added processed products aiding enhanced shelf-life and better product quality; however, certain processing steps impact negatively by enriching the level pesticide residues in the final product by developing toxic metabolites or second- and third-generation derivatives. Post-harvesting operations such as washing, peeling, chopping, etc. help in reducing the pesticides on the surface of fruit and vegetable commodities (Yigit and Velioglu, 2020). The heat treatments such as pasteurization, sterilization, blanching, frying, boiling, cooking, etc. help in the reduction of pesticides by chemical reactions due to oxidation and hydrolysis of chemical compounds. Also, low moisture content, pH, and time–temperature combination during cooking also modulate the residual pesticide limit in the final product. Similarly, unit operations such as drying and grinding of samples, canning of food products, etc. abundantly reduce the residual limits by evaporating water and altering the physico–chemical nature of pesticides (Kaushik et al., 2009). However, the unit processing operations such as cereal grain processing, fruit processing, oil extraction, grape, egg drying, and so on have a high risk of increased levels of residual pesticides and are affected by a variety of factors such as the physico–chemical behavior of pesticide molecules, produced metabolites during the chemical process, their photostability, lipophilicity, thermal stability, and polarity (Scholz et al., 2017).

## Determination of pesticide residues in food matrix

The determination of the residual pesticide limit in RTE/RTS foods involves a complex phenomenon and requires some special criteria. The extractability of a pesticide residue depends on the biochemical nature and behavior of food. The complexity of a matrix behavior is often increased by the processing operations involved, which impacts the performance method by decreasing precision as well as accuracy. Therefore, usage of matrix-matched calibrations and selective clean-up practices are necessary to avoid such issues (Law et al., 2019). The worldwide harmonization of maximum residual limits (MRLs) for pesticide residues in raw agricultural commodities has attained a high recognition. Similarly, in India, food technologists and central agency such as Food Safety and Standard Authority of India (FSSAI) are now emphasizing too much toward a sustainable growth in the processed food sector for making and consumption of value-added items with safe or lower residual limits of pesticides (Muralidhara et al., 2022).

## Eco-friendly management of pesticides as bioremediation

Physical and chemical cleaning of pesticides release more toxic compounds, and both are harmful as well as costly. To maintain a sustainable environment with a healthy and productive ecosystem, eco-friendly approach as bioremediation methods is available to remove harmful contaminants (Desisa et al., 2022). Since plants, algae, fungi, bacteria, and their interactions are used to remove toxins *via* bioremediation, which serves as a cost-effective and environmentally benign method. Pesticide remediation today includes a variety of environment friendly techniques, such as phytoremediation, microalgae bioremediation, myco-remediation, and bacterial pesticide degradation (Singh et al., 2020).

Phytoremediation is an economical, solar-powered method that involves the removal or reduction of harmful chemicals from damaged sites using effective plant species. *Kochia* sp., *Triticum* spp., *Ricinus communis* and *Ceratophyllum demersum* are well-known plant species that have played a significant role in the removal of atrazine, lindane, chlorpyrifos, and endrin, respectively. The absorption of pesticides by plants results in the conversion of hazardous pesticides into less toxic compounds, which helps to remove toxic pollutants from polluted sites. Plants use various mechanisms to remove pollutants, including pollutant transpiration (phytovolatilization), clean-up through the rhizosphere microbiome (rhizo-degradation), enzymatic degradation (phytodegradation), and pesticide accumulation in different plant parts (phytoextraction). Such plants also improve the landscapes, reduce soil erosion, and prevent pollutant seepage. In addition, phytoremediation serves as an economic, safe, and green approach for chemical waste treatment (Subashini et al., 2007; Gill and Garg, 2014; Mishra et al., 2015; Rissato et al., 2015; Kuppusamy et al., 2016; Main et al., 2017; Mir et al., 2017; Koranteng et al., 2018; Perez-Lucas et al., 2018; Singh et al., 2020).

Microalgae are also known as effective biosorbents of heavy metals and pesticides and can remove them from contaminated areas. *Chlamydomonas reinhardtii*, *Chlamydomonas mexicana*, and *Dunaliella* sp. have been reported for the removal of prometryne, atrazine, and mirex pesticides, respectively. Such photoautotrophic organisms exist in different forms in nature and are involved in the conversion of radiant energy (light energy to chemical energy). The use of microalgae results in the production of oxygen, which preserves the environment’s balance. Oxygen generated from microalgae also helps the bacteria during the biodegradation process. Microalgae have been found to use chemical pollutants as an energy alternate and to accelerate the biodegradation process. It can be used to achieve a variety of objectives, including nutrient recovery from wastewater, biomass formation, removal of contaminants (bioaccumulation and biosorption), and being able to grow under stress conditions. In which, bioaccumulation is an energy-dependent active process involving living organisms that metabolize pollutants. Whereas biosorption is an energy-independent process that involves both dead and living organisms for the removal of contaminant form polluted environments. The use of such technology in a two-way manner, such as pesticide accumulation as well as conversion of toxic into less toxic compounds. The degradation is influenced by the introduction of potent microalgae, optimum conditions, and the chemical composition of pesticides. In addition, there are some major factors that alter the degradation process of pesticides, such as molecular weight, functional group, concentration, and water solubility. Under stress conditions, these microalgae act mixotropically and derive their energy from light and organic carbon, which gives them an advantage over bacteria and fungi during biodegradation (Velasquez and Dussan, 2009; Chojnacka, 2010; Mata et al., 2010; John et al., 2011; Subashchandrabose et al., 2011; Monteiro et al., 2012; Rath, 2012; Chekroun et al., 2014; Kabra et al., 2014; Torres et al., 2017; Singh et al., 2020).

Myco-remediation is another type of biological approach to pesticide waste management, where fungi can use such pollutants as a carbon source and convert them into less toxic compounds, thus cleaning them from the water and soil system. Fungi are ideal among microorganisms due to their structural morphology, which contains hyphae, that allows the transfer of small chemical molecules by microscopic pores easily. The mycelium networks have a multi-functional role, in addition to accelerating pesticide degradation, they also improve the plant’s nutrient and water availability. Ligninolytic fungi are known to secrete a variety of extracellular enzymes that aid in the transformation of recalcitrant chemical compounds. While saprotrophic fungi excrete the most enzymes, followed by other fungi (soft rot, white rot, and brown rot). White-rot fungi (*P. Pleurotus ostreatus*, *Trametes hirsutus*, and *Cyathus bulleri*) are widely known for pesticide biodegradation due to their extracellular enzyme complex (e.g., laccase, manganese peroxidase, and lignin peroxidase) acting non-specifically. The consortium of potent fungal species was found to be suitable for chlorpyrifos and DDT biodegradation. The phyla Zygomycota, Ascomycota, and Basidiomycota are reported for biodegradation *via* attacking on functional groups (dehydrogenation, demethylation, hydroxylation, etc.). This process is also influenced by other factors such as optimal temperature, pH, moisture, nutrient, and water availability, all of which play a significant role in pesticide degradation. Nowadays, many developing countries cannot afford biopesticides or cannot avoid the use of chemical pesticides, so they need to use myco-remediation or other bioremediation approaches to control pesticide pollution in a parallel manner (Tortella et al., 2005; Huang et al., 2008; Sagar and Singh, 2011; Adenipekun and Lawal, 2012; Chen et al., 2012; Wu et al., 2015; Maqbool et al., 2016; Janusz et al., 2017; Singh et al., 2020).

Bacteria have been widely reported to degrade and remove pesticides as compared to other remedial approaches. *Pseudomonas*, *Azotobacter*, *Flavobacterium*, and *Arthrobacter* are the major bacterial genus involved in the removal of pesticides from polluted environments. The discovery of pollutant-degrading bacteria aided by advances in genetic engineering methods. These microbes use the pesticide for nutrients, generate H_2_O and CO_2_, and overcome the environmental risk associated with pesticides. In the soil system, such pesticides accumulate and act as electron donors and carbon sources for soil microorganisms. The environmental conditions, pesticide exposure time, and concentration, bacterial type, and growth factors (such as temperature, pH, moisture, nutrient, and water availability) all are important for efficient biodegradation. However, the presence of sulfate and chloride act as anion and bind strongly to microbes that blocks the microbial action on pesticides. The chemical structure is the first target of microbial degradation and converted into inorganic components that are further utilized by the microorganism. Advanced approaches such as bioaugmentation, bio-stimulation and natural attenuation are employed to increase the pesticide biodegradability, which includes potent bacteria, nutrient addition, and the introduction of native species to the contaminated site respectively. *Alcaligenes, Flavobacterium, Acinetobacter* are reported as endosulfan degrading bacteria. Similarly, *Stenotrophomonas* sp. also known for almost 100% removal of diazinon from the contaminated site. The bacterial system is well studied as compared to other bioremediation technologies. The diverse bacterial groups and their corresponding enzymes responsible for degradation are explained in the “Biodegradation of Pesticide Pollutants” section (Gavrilescu, 2005; Singh and Walker, 2006; Arias-Estevez et al., 2008; Huang et al., 2008; Singh et al., 2011, 2020; Laura et al., 2013; Rani and Dhania, 2014; Adams et al., 2015; Deng et al., 2015).

## Biodegradation of pesticide pollutants

Biodegradation of pesticides is mainly mediated by using microbial systems. Microbes are able to produce a specific group of enzymes that are able to catalyze the pesticides from contaminated sites. The pure culture and mixed cultures of the bacteria and fungi were found to be effective in the removal of pesticide residues from the water and soil environment. Microbial consortium was found with superior degradation abilities (Bhatt et al., 2021c). Singh et al. (1999) found that microbes have developed a number of metabolic routes to breakdown or detoxify a variety of environmental contaminants, including pesticides. Conde-Avila et al. (2021) reported bacteria from the genera *Streptomyces*, *Flavimonas*, *Burkholderia*, *Micrococcus*, *Sphingomonas*, *Brevibacterium*, *Flavobacterium*, *Pseudomonas*, *Agrobacterium*, *Arthrobacter*, *Enterobacter*, and *Bacillus* are associated with pesticide biodegradation. There is a diverse group of bacteria and fungi that are capable of degrading pesticides. The different phyla include Bacteroidetes, Basidiomycota, Chlorophyta, Cyanobacteria, Actinomycetota, Firmicutes, and Proteobacteria. The bacteria that fall under Actinobacteria have a tremendous capability to degrade several classes of chemical pesticides as most of the strains have high GC content and are actively used for the recycling of complex polymers. *Streptomyces*, *Nocardioides*, *Arthrobacter*, *Rhodococcus*, *Micrococcus*, and *Microbacterium* are members of the *Actinomycetota* phylum and can metabolize a variety of chemical compounds such as organochlorides, organophosphates, carbamates, triazinones, and others (Kim et al., 2017). Similarly, Firmicutes are also play a critical role in pesticide biodegradation. Among them, several strains possess endospores that are resistant to any adverse condition and are reported as extremophiles. There are a number of firmicutes that are capable of degrading pesticides, including *Paenibacillus polymyxa, Bacillus licheniformis*, *Bacillus thuringiensis*, *Bacillus pumilus*, *Bacillus subtilis*, and *Bacillus cereus* (Patil et al., 1970). Moreover, among the proteobacteria, α-, β-, and γ-proteobacteria have also been reported for their pesticide degradation activity.

Among the α-proteobacteria strains that have been reported are *Sphingomonas*, *Rhizobium*, *Methylobacterium*, *Azospirillum*, *Pseudaminobacter*, *Bosea*, *Mesorhizobium*, *Shinella*, and *Ochrobactrum*. Moreover, *Ralstonia*, *Alcaligenes*, *Burkholderia*, *Achromobacter*, and *Cupriavidus* are the reported bacterial strains among β-proteobacteria. Furthermore, reported bacterial strains among γ-proteobacteria are *Yersinia*, *Pseudomonas*, *Klebsiella*, *Acinetobacter*, *Serratia*, and *Xanthomonas* (Bhatt et al., 2020b; Kumar et al., 2021). Microbes and their enzymes associated with biodegradation of different types of pesticide are shown in Tables 6, 7.

**TABLE 6:** Pesticides degrading microorganisms.

Type of pesticide	Example	Microorganism	References
Organophosphorus	Chlorpyrifos	*Bacillus*spp., *Pseudomonas*spp., *Arthrobacter*spp., *Micrococcus, Flavobacterium, Bacillus licheniformis, Cupriavidus*spp., *Burkholderia caryophylli, Brevundimonas diminuta, Spirulina platensis, Synechocystis*	Nandhini et al., 2021; Lourthuraj et al., 2022
Organophosphorus	Parathion	*Pseudomonas diminuta, Flavobacterium*spp., *Pseudomonas stutzeri, Arthrobacter*spp., *Agrobacterium radiobacter, Bacillus*spp., *Xanthomonas*spp.	Mali et al., 2022; Saravanan et al., 2022
Organophosphorus	Methyl parathion	*Pseudomonas*spp., *Bacillus*spp., *Plesiomonas*spp., *Pseudomonas putida*,	Dong et al., 2005; Singh and Walker, 2006; Parakhia et al., 2014
	Glyphosate	*Pseudomonas*spp., *Alcaligene*spp., *Bacillus megaterium, Rhizobium*spp., *Agrobacterium*spp., *Arthrobacter atrocyaneus, Geobacillus caldoxylosilyticus*,	Huch et al., 2022; Zhang et al., 2022
Organophosphorus	Coumaphos	*Nocardiodes simplex, Agrobacterium radiobacter, Pseudomonas diminuta, Pseudomonas monteilli, Flavobacterium*spp., *Nocardiodes Strain B-1*	Singh and Walker, 2006; Blatchford et al., 2012
Organophosphorus	Monocrotophos	*Pseudomonas*spp., *Bacillus subtilis, Arthrobacter*spp., *Peudomonas mendocina, Bacillus megaterium, Arthrobacter atrocyaneus, Pseudomonas aeruginosa, Clavibacter michiganense*	Kaur and Goyal, 2019
Organophosphorus	Fenitrothion	*Flavobacterium*spp., *Arthrobacter aurescenes; Burkholderia*spp.	Singh and Walker, 2006; Hong et al., 2007
Organophosphorus	Fenthion	*Bacillus*spp.	Aislabie and Lloyd-Jones, 1995
Organophosphorus	Diazinon	*Flavobacterium*spp., *Pseudomonas*spp., *Arthrobacter*spp.	Aislabie and Lloyd-Jones, 1995; Singh and Walker, 2006
Organophosphorus	DDT	*Alcaligene eutrophus*	Aislabie and Lloyd-Jones, 1995
Organochlorine	Aldrin	*Micrococcus, Bacillus polymyxa, Flavobacteria, Pseudomonas fluorescens, Phlebia aurea, Phlebia acanthocystis, Phlebia brevispora*	Bose et al., 2021
Organochlorine	Dieldrin	*Pseudomonas fluorescens, Phlebia aurea, Phlebia acanthocystis, Phlebia brevispora*	Bose et al., 2021
Organochlorine	Endosulfan	*Pseudomonas aeruginosa, Pseudomonas fluorescens, Mortierella*sp.,*Trametes hirsute, Aspergillus niger*	Romero-Aguilar et al., 2014; Bose et al., 2021
Organochlorine	Alpha endosulfan	*Fusarium ventricosum, Klebsiella, Acinetobacter*	Siddique et al., 2003; Bose et al., 2021
Organochlorine	Beta endosulfan	*Fusarium ventricosum*	Siddique et al., 2003
Organochlorine	Dichlorodiphenyl-trichloroethane	*Trichoderma harzianum, Stenotrophomonas*sp., *Sphingobacterium*sp., *Pseudomonas*sp., *Trichoderma hamatum, Rhizopus arrhizus*,	Ortiz-Hernández et al., 2013; Russo et al., 2019; Bose et al., 2021
Organochlorine	Lindane	*Microbacterium* sp. P27, *Paracoccus* sp. NITDBR1, *Streptomyces*sp. A5,*Streptomyces*sp. M7, *Pleurotus eryngii, Pleurotus florida, Pleurotus sajor-caju*, *Phanerochaete chrysosporium*	Rigas et al., 2005; Xiao and Kondo, 2020; Zhang W. et al., 2020
Triazone	Atrazine	*Nocardia*spp.*Pseudomonas*spp., *Rhodococcus*spp.	Aislabie and Lloyd-Jones, 1995
Carbamate	Carbafuron	*Achromobacter*spp., *Pseudomonas*spp., *Flavobacterium*spp.	Aislabie and Lloyd-Jones, 1995
	EPTC	*Arthrobacter*spp., *Rhodococcus*spp.	Aislabie and Lloyd-Jones, 1995
	Carbafuron	*Achromobacter spanius, Diaphorobacter polyhydroxybutyrativorans*	Rahman et al., 2018
Avermectin	Emamectin Benzoate	*Achromobacter spanius, Diaphorobacter polyhydroxybutyrativorans*	Rahman et al., 2018
Neonicotinoid	Thiamethoxam	*Achromobacter spanius, Diaphorobacter polyhydroxybutyrativorans*	Rahman et al., 2018

**TABLE 7:** Bacterial enzymes, responsible for the degradation of pesticides (Ortiz-Hernández et al., 2013).

Pesticide	Enzyme	Bacteria
Gylphosate	Oxidoreductase (Gox)	*Pseudomonas*spp.,*Agrobacterium* spp.
Endosulfan, aldrin, malathion, DDT, endosulfate	Monooxygenases (Esd)	*Mycobacterium*spp.,*Arthrobacter* spp.
Hexachlorobenzene, Pentachlorobenzene	P450	*Pseudomonas putida*
Trifluralin	Dioxygenases (TOD)	*Pseudomonas putida*
Hexachlorocyclohexane	Haloalkane Dehalogenases (Lin B)	*Sphingobium*spp.
Chloro-S-trazina	AtzA	*Pseudomonas*spp.
Chloro-S-trazina	TrzN	*Nocardioides*spp.
Hexachlorocyclohexane (Gamma isomer)	Lin A	*Sphingobium*spp.
2,4-dichlorophenoxyacetic acid	TfdA	*Ralstonia eutropa*
Pyridyl-oxyacetic acid	TfdA	*Ralstonia eutropa*
Pyridyl-oxyacetic acid	DMO	*Pseudomonas maltophilia*
Phosphotriester	Phosphotriesterases (OPH/OpdA)	*Pseudomonas diminuta, Agrobacterium radiobacter, Flavobacterium*spp.

The basic stages of pesticide conversion were characterized by Kumar et al., 1996 as follows: (1) Mineralization: Carbon dioxide or methane as an end-product of complete degradation; (2) Detoxification: Conversion of toxic to non-toxic compounds; (3) Co-metabolism: Microbes involved in the metabolism process of compounds without benefiting themselves from these compounds; (4) Activation: Activation of compounds. During the beginning of 1064, hydrolases and oxygenases came in knowledge and Singh et al. also reported involvement of these enzymes in pesticide biodegradation (Bollag et al., 1968; Tiedje et al., 1969; Singh et al., 1999). Under both denitrifying and aerobic conditions, hydrolytic dehalogenation (the substitution of a halogen group by a hydroxyl) can occur, but only methanogenic and sulfonic circumstances result in reductive dehalogenation, which involves the substitution of a halogen group by a hydrogen group. Furthermore, biotransformation events such as polymerization and methylation may occur, resulting in more hazardous or recalcitrant compounds. Different methods for converting hazardous pesticides were used, depending on their chemical constituents and the microbes that were used for bioconversion (Singh et al., 1999). Factors such as the microbial culture, cultivation technique, size of inoculum, growth under elevated pesticide percentage, adaptation, rhizosphere interactions, and response against the environmental factors can all affect the pesticide degradation process (Conde-Avila et al., 2021). Research has concentrated on the practice of microbial cellular immobilization (CI) technology in several materials and supports the long-lasting survival of microbes. Now, research has shifted to the use of microbial cells as CI, which protects and allows them to be reused. Such a strategy enhances the possibilities of techniques lasting and succeeding in a pesticide-contaminated environment for a long period and has been found suitable for pesticide biodegradation (Colla et al., 2014; Pradeep and Subbaiah, 2016; Fernández-López et al., 2017; Conde-Avila et al., 2021).

The CI technology has served as an environmentally approachable processes for waste management practices. The use of CI of degrading microbes in the elimination and or degradation of pollutants, the CI system has developed as an eco-friendly alternative approach. There are certain disadvantages to CI technology, such as microbial interactions with the immobilization material and its impact on microbial survivability (Conde-Avila et al., 2021). When CI is utilized instead of free cells, the percentage of clearance and efficiency increases for pesticides including chlorpyrifos, atrazine, difenoconazole cypermethrin, carbaryl, endosulfan, and carbofuran. The benefits of utilizing CI are independently supported by the immobilization method or substance employed (Bhadbhade et al., 2002; Pattanasupong et al., 2004; Adinarayana et al., 2005; López-Pérez et al., 2006; Fuentes et al., 2013; Zucca and Sanjust, 2014; Abigail and Das, 2015; Chen et al., 2015; Tallur et al., 2015; Bhatt et al., 2016; Fernández-López et al., 2017). Because pesticides come in such a wide variety of chemical groups, the factors that influence their presence, transit, and mobility are complicated and difficult to anticipate. Extrinsic and intrinsic variables govern adsorption-desorption, biodegradation, volatilization, photodegradation and breakdown phenomena, which mediate pesticide occurrence, and migration (Conde-Avila et al., 2021). Soils with high organic matter reduce pesticide availability through adsorption to a larger percentage than sandy soils (Yanez-Ocampo et al., 2016).

Bioremediation procedures frequently include organic wastes and/or specialist strains with catabolic capabilities against contaminants to assist the breakdown of more persistent pesticides or to reduce their influence on microorganisms. Using genetically engineered strains to breakdown pesticides might be an effective method. Pesticide-exposed native species can develop the capacity to degrade toxic chemicals. Such technology was created to clean up pesticide-related pollutants (Barreiros et al., 2012; Nikel et al., 2014; Castillo et al., 2016; Bhatt et al., 2019a,b,c, 2020c, 2021d).

In comparison to pure cultures, the introduction of consortia or pesticide primed materials has been found to improve pesticide breakdown and mineralization capability in BPSs (bio-purification systems) (Sniegowski and Springael, 2015). Furthermore, Biobed bioremediation systems can be an ideal microcosm for developing specialized microorganisms capable of enhancing pesticide residue metabolization from wastewaters (Dunon et al., 2013). However, the bioaugmentation strategy for various pesticide biodegradations in wastewaters at high concentrations, as occurs in real-world scenarios, is still little known (Sniegowski and Springael, 2015).

## Conclusion

Pesticide use has expanded extensively in the recent years, resulting in the environmental damage, particularly water and soil contamination. Pesticides come in a variety of forms, but organophosphates, organochlorine, carbamate, and pyrethroids are the most abundantly uses pesticides and have human and environmental concerns. Refined knowledge of various properties related to the physical and chemical background of pesticides are necessary to determine the impact and behavior of pesticide transformation in that environment. Such pesticides need proper management strategies for converting them to non-toxic compounds before releasing them into the environment. They are the most persistent and generally resistant to degradation under natural conditions. The scientific community has been working hard to come up with creative approaches to pesticide pollution reduction. Environmentally friendly management strategies include several bioremediation approaches and servers to solve pesticide problems or develop alternative green solutions. Bioremediation strategies such as phytoremediation, microalgae bioremediation, myco-remediation, and microbial degradation are also cost-effective and environmentally benign methods. Nowadays, microbial degradation methods are used extensively. Microorganisms and their enzymes play a key role in the breakdown of chemical compounds and synthetic pesticides. Although these methods are environmentally friendly, they have certain limitation such as metabolic routes followed by microbes are highly influenced by external factors. As a result, further study is needed in specific areas before this approach can be declared successful. Enzymatic degradation appears to be a viable method. It is becoming increasingly vital to do significant research to find enzymes capable of degrading synthetic pesticides. Microbial degradation occurs at a considerably slower rate and is not always as efficient or straightforward to carry out as traditional bioremediation technologies. It needed to find more potent microbes, novel genes, and bioremediation approaches for proper waste management of pesticide pollutants. Genetically engineered microorganisms and biotechnology also play a significant role in this area. The above discussion illustrates the utilization of pesticide degrading microorganisms in a constructive way to manage the pesticide pollutants in an eco-friendly manner. Hence, the further studies on the screening of effective microbial strains and enzymes are essential to reduce pesticide risks for the environment and human health.

## Author contributions

VMP: conception and design of study and revising the manuscript critically for important intellectual content, approval of the version of the manuscript to be published. VMP and VV: analysis and/or interpretation of data. VMP, VV, BR, BK, NB, AS, SD, MY, RK, SS, AM, VP, NR, and JC: acquisition of data. VMP, BR, and BK: drafting the manuscript. All authors approved the version of the manuscript to be published.
